# Optimization of chondrocyte isolation from human articular cartilage to preserve the chondrocyte transcriptome

**DOI:** 10.3389/fbioe.2022.1046127

**Published:** 2022-11-21

**Authors:** Ping Shen, Peihua Wu, Tazio Maleitzke, Marie-Jacqueline Reisener, Gitta A. Heinz, Frederik Heinrich, Pawel Durek, Clemens Gwinner, Tobias Winkler, Matthias Pumberger, Carsten Perka, Mir-Farzin Mashreghi, Max Löhning

**Affiliations:** ^1^ Pitzer Laboratory of Osteoarthritis Research, German Rheumatism Research Center (DRFZ), A Leibniz Institute, Berlin, Germany; ^2^ Department of Rheumatology and Clinical Immunology, Charité—Universitätsmedizin Berlin, Corporate Member of Freie Universität Berlin and Humboldt-Universität zu Berlin, Berlin, Germany; ^3^ Stem Cell and Biotherapy Engineering Research Center of Henan Province, College of Life Sciences and Technology, Xinxiang Medical University, Xinxiang, China; ^4^ Center for Musculoskeletal Surgery, Charité – Universitätsmedizin Berlin, corporate member of Freie Universität Berlin and Humboldt-Universität zu Berlin, Berlin, Germany; ^5^ Julius Wolff Institute, Berlin Institute of Health at Charité – Universitätsmedizin Berlin, Berlin, Germany; ^6^ BIH Charité Clinician Scientist Program, BIH Biomedical Innovation Academy, Berlin Institute of Health at Charité – Universitätsmedizin Berlin, Berlin, Germany; ^7^ German Rheumatism Research Center (DRFZ), a Leibniz Institute, Berlin, Germany

**Keywords:** chondrocyte isolation, human articular cartilage, small chondrocytes, large chondrocytes, transcriptome preservation, single-cell RNA sequencing

## Abstract

The isolation of chondrocytes from human articular cartilage for single-cell RNA sequencing requires extensive and prolonged tissue digestion at 37 C. Modulations of the transcriptional activity likely take place during this period such that the transcriptomes of isolated human chondrocytes no longer match their original status *in vivo*. Here, we optimized the human chondrocyte isolation procedure to maximally preserve the *in vivo* transcriptome. Cartilage tissues were transferred into a hypoxia chamber (4% O_2_) immediately after being removed from OA patients and minced finely. Collagenase II at concentrations of 0.02%, 0.1%, 0.25%, 0.5%, 1%, and 2% was applied for 0.5, 1, 2, 4, and 18 h to digest the minced tissue. Actinomycin D (ActD) was added to test its capacity in stabilizing the transcriptome. Cell yield, viability, cell size, and transcriptome were determined using counter chamber, flow cytometry, and RNA sequencing (RNA-seq). Collagenase II at 2% concentration released small chondrocytes from cartilage matrix during the first digestion hour and started to release large cells thereafter, reaching a complete release at 4 h. During 4-h digestions, collagenase II at 2% and 1% but not at lower concentrations yielded maximal release also of the large chondrocyte population. RNA-seq analysis revealed that a 4-h digestion period with 1% or 2% collagenase II plus Actinomycin D optimally preserved the transcriptome. Thus, this study provides an isolation protocol for single chondrocytes from human articular cartilage optimized for transcriptome preservation and RNA-seq analysis.

## Introduction

Osteoarthritis (OA) is a chronic degenerative joint disease hallmarked by articular cartilage breakdown, which affects more than 300 million people worldwide (Safiri et al., 2020). Chondrocyte dysfunction is a key driver of OA pathology (Hwang and Kim, 2015). Hence, characterization and comparison of chondrocytes derived from healthy and diseased donors can provide insight into chondrocyte pathology. This may help to develop strategies to restore normal chondrocyte function. However, chondrocytes are sparsely distributed in human articular cartilage and surrounded by a dense network of extracellular matrix (ECM). The ECM is principally composed of collagen fibrils, proteoglycans, and other non-collagenous proteins and glycoproteins (Sophia Fox et al., 2009). Thus, isolating chondrocytes from human articular cartilage requires lengthy enzymatic digestion, which is usually performed at 37°C, because at 27 C it yielded a significantly lower number of chondrocytes (Hidvegi et al., 2006). Most of the digestion protocols apply a two-step digestion process involving various enzymes (Kuhne et al., 2009; Oseni et al., 2013). Pre-digestion using trypsin, dispase, pronase, or hyaluronidase serves to soften the hard cartilage tissue to allow better access of collagenase to the collagen fibrillar network. Then collagenase II digestion releases the embedded cells. Nevertheless, also one-step digestion with only collagenase II targeting the extensive collagen network, has been applied with no apparent disadvantage in the efficiency of obtaining live cells (Manning and Bonner, 1967; Barbero et al., 2004; Tew et al., 2008; Yonenaga et al., 2010). However, the concentrations and incubation times of collagenase II vary significantly between the protocols published, ranging from 0.05% to 2% (w/v concentration) at a duration range from 2 to 24 h (Barbero et al., 2004; Yonenaga et al., 2017). Despite variations in chondrocyte isolation protocols, the general yield of cells reproducibly remains at 2–4 million per gram articular cartilage tissue for individuals aged between 40 and 90°years (Barbero et al., 2004).

Lengthy tissue digestion at 37 C most likely alters the transcriptome of the cells (Westendorf et al., 2014), such that in the end, the transcriptomes of chondrocytes isolated *ex vivo* do not faithfully reflect the transcriptional status of the cells *in vivo*. This is particularly problematic for RNA-seq, especially single-cell RNA-seq analysis. To date, optimizations of chondrocyte isolation have focused on the maximization of cell yields and viability. A systematic study to optimize the isolation procedure towards best preservation of the *in vivo* transcriptomes is lacking. We conducted this study to optimize the isolation procedure to obtain chondrocytes for transcriptome-sensitive analyses, such as RNA-seq, especially single-cell RNA-seq analysis.

## Materials and equipment

### Materials

• 15 cm Petri dish (CELLSTAR; Cat #639160)
• scalpel (Braun; Cat #BA824SU)
• 50 ml Falcon tubes (SARSTEDT; Cat #62.559.001)
• 15 ml Falcon tubes (SARSTEDT; Cat #62.554.502)
• Micro tubes 1.5 ml (SARSTEDT; Cat #72.706.400)

### Reagents

• DPBS (Gibco; Cat #14190-144)
• DMEM/F-12 medium (Gibco; Cat #330-038)
• Collagenase II (Biochrom, Cat#C2-22; Gibco, Cat #17101-015; StemCell™; Cat #07418; Sigma; Cat #Cas9001-12-0)
• Actinomycin D (Merck; Cat A1410)
• Propidium iodide (PI)/Annexin V staining kit (Thermo Fisher; Cat #A35110);
• QIAzol Lysis Reagent (Qiagen; Cat #79306)
• RNeasy Mini Kit (Qiagen; Cat 217004)

### Equipment

• Miltenyi Biotec MACSmix Tube Rotator (Cat #130-090-753)
• Hypoxia hood and incubator (BioSpherix, X3 Xvivo System)
• FACSCanto II (Becton Dickinson)
• Centrifuge (Thermo Scientific)

## Methods

### Chondrocyte isolation

Full-thickness cartilage was surgically removed from the femoral condyles of osteoarthritic patients (25 patients; mean age 67.4 years; female:male ratio = 4:1) who underwent total knee arthroplasty performed at the Center for Musculoskeletal Surgery of Charité—Universitätsmedizin Berlin. Cartilage tissues were transferred into a hypoxia chamber (4% O_2_) and minced finely for chondrocyte isolation. For enzymatic digestion, minced cartilage was incubated in 10 ml collagenase II solution at concentrations of 0.02%, 0.1%, 0.25%, 0.5%, 1%, or 2% for 0.5, 1, 2, 4, or 18 h, with or without Actinomycin D (ActD, 2 μg/ml) at 37 °C and continuous rotating at 20 rpm. Subsequently, the digestion solution was filtered through 70 μm cell strainer and cells were washed. Cell yields were determined using cell counter chamber; cell viability and size were detected by flow cytometry (Propidium iodide (PI) or Zombie dye staining). Cell transcriptomes were determined by RNA-seq.

### Flow cytometry analysis

Chondrocyte were stained with PI and/or Annexin V and then acquired on a FACSCanto II (Becton Dickinson). Data were analyzed with Flow Jo (version 10.6.2).

### RNA isolation and RNA-seq

Fresh cartilage tissue was minced finely in a hypoxia chamber, transferred into a plastic bag, and snap frozen in liquid nitrogen. Cartilage pieces were further pulverized before being transferred into Trizol buffer and blended using Ultra Turrax (IKA T10 basic). Chondrocytes collected by using various digestion protocols were also lysed in Trizol buffer. Total RNA was then extracted using the RNeasy Mini Kit (Qiagen; 217,004). RNA integrity was assessed using a Fragment analyzer (Agilent), and cDNA libraries were generated for samples with high RNA integrity (RQN >8), using the Smart-Seq v4 mRNA Ultra Low Input RNA Kit (Takara Bio) with up to 10 ng of RNA according to manufacturer’s instructions. Paired-end sequencing (2 × 75 bp) of cDNA libraries was performed on an Illumina NextSeq500 device. Obtained reads were mapped to the hg19 genome (annotation releases: GRCh37. p13) using Tophat261 and Bowtie262 with very-sensitive settings. Read counts were determined with featureCounts63. Further analysis was performed using R (4.0.3).

### Statistical analysis

Statistical analysis was performed using GraphPad Prism (v5.02 and v7) software. Statistical significance was determined by paired or unpaired two-tailed *t*-test for two-group comparison and One-way ANOVA for multiple comparison.

## Results

### Collagenase II from four suppliers performed similarly well with only slight differences in isolating viable chondrocytes from human articular cartilage

**FIGURE 1:**
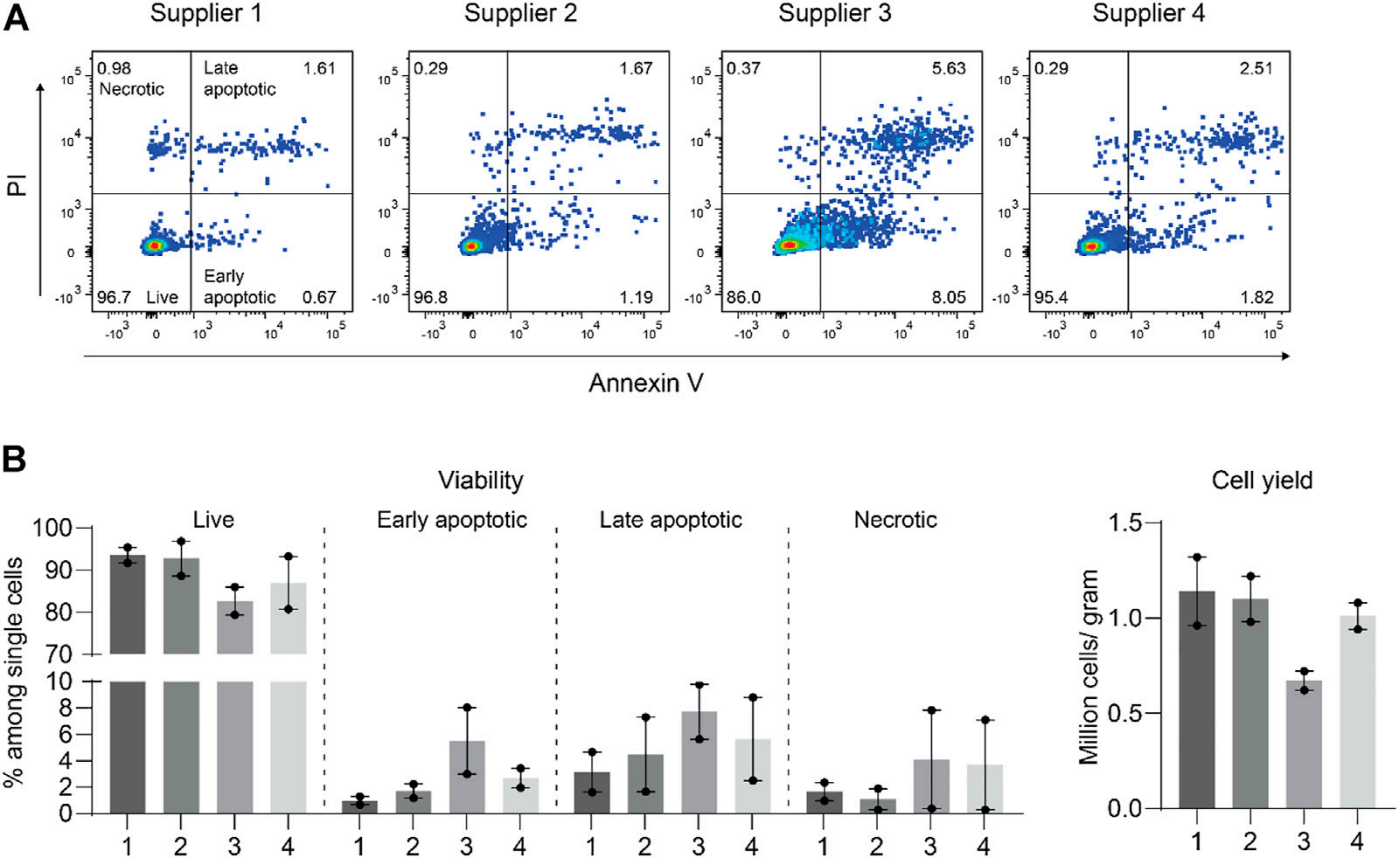
Collagenase II from four suppliers performed similarly well with only slight differences in isolating viable chondrocytes from human articular cartilage. **(A)** Apoptotic and necrotic states were determined by propidium iodide (PI) and Annexin V staining of chondrocytes released from human articular cartilage tissue by digestion with collagenase II obtained from four different commercial suppliers. Representative dot plots from FACS analysis are shown. **(B)** Percentages of live (Annexin V− PI−), early apoptotic (Annexin V+ PI−), late apoptotic (Annexin V+ PI+), and necrotic cells (Annexin V− PI+) and total numbers of recovered cells per gram of cartilage tissue are plotted. Data were generated from two independent experiments with samples from two individual patients.

Collagenase II from *Clostridium* histolyticum is widely used for the disaggregation of cartilage and is provided by various suppliers (Xia et al., 2013). We first tested collagenase II from four different suppliers by incubating them individually at a concentration of 0.1% with an equal amount of minced cartilage tissue of the same patients. After an 18-h digestion when no visible cartilage pieces remained, we filtered the solution and determined the total cell number and cell viability. Collagenase II from supplier 1, 2, and 4 was found to be similar and slightly better than that from supplier three in obtaining viable chondrocytes (Figures 1A,B). Thereafter, collagenase II of supplier one from the same lot was stored and used throughout the study.

### Enzymatic digestion of cartilage yields increasing cell numbers with extended digestion time for up to 4 h

**FIGURE 2:**
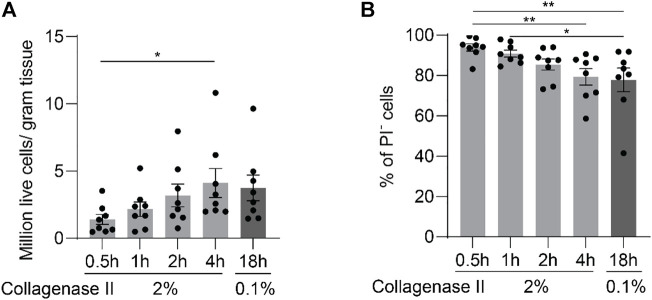
Enzymatic digestion of cartilage yields increasing cell numbers with extended digestion time for up to 4 h. After being finely minced, human articular cartilage tissue was divided equally into five portions, and the samples were digested with 2% collagenase II for 0.5, 1, 2, or 4 h; or with 0.1% collagenase II for 18 h. Cell yields **(A)** and viability as quantified by FACS **(B)** at the end of each digestion protocol are plotted (n = 8 patients). Data were analyzed using paired One-way ANOVA (*p < 0.05, **p < 0.01).

Modulations of the transcriptional activity likely take place during extended digestion periods at 37 C. In order to obtain chondrocytes in the shortest possible digestion time, we increased the concentration of collagenase II to 2% and started to harvest cells after incubation for 0.5, 1, 2, and 4 h. These digestion protocols are designated in short: 2%-0.5h, 2%-1h, 2%-2h, and 2%-4 h. The digestion with 0.1% collagenase II for 18 h (0.1%-18 h) always leads to a complete digestion and is also the most often used digestion protocol in the field. Thus it was used as a reference. Total cell number and cell viability were determined at the end of each incubation period (Figures 2A,B). Of note, complete digestion of the minced tissue was achieved only by the protocols 0.1%-18 h and 2%-4h, which was reflected in comparable and peak cell recovery rates.

### Chondrocyte transcriptomes from 2- and 4-h cartilage digests most closely resemble those obtained directly *ex vivo*

**FIGURE 3:**
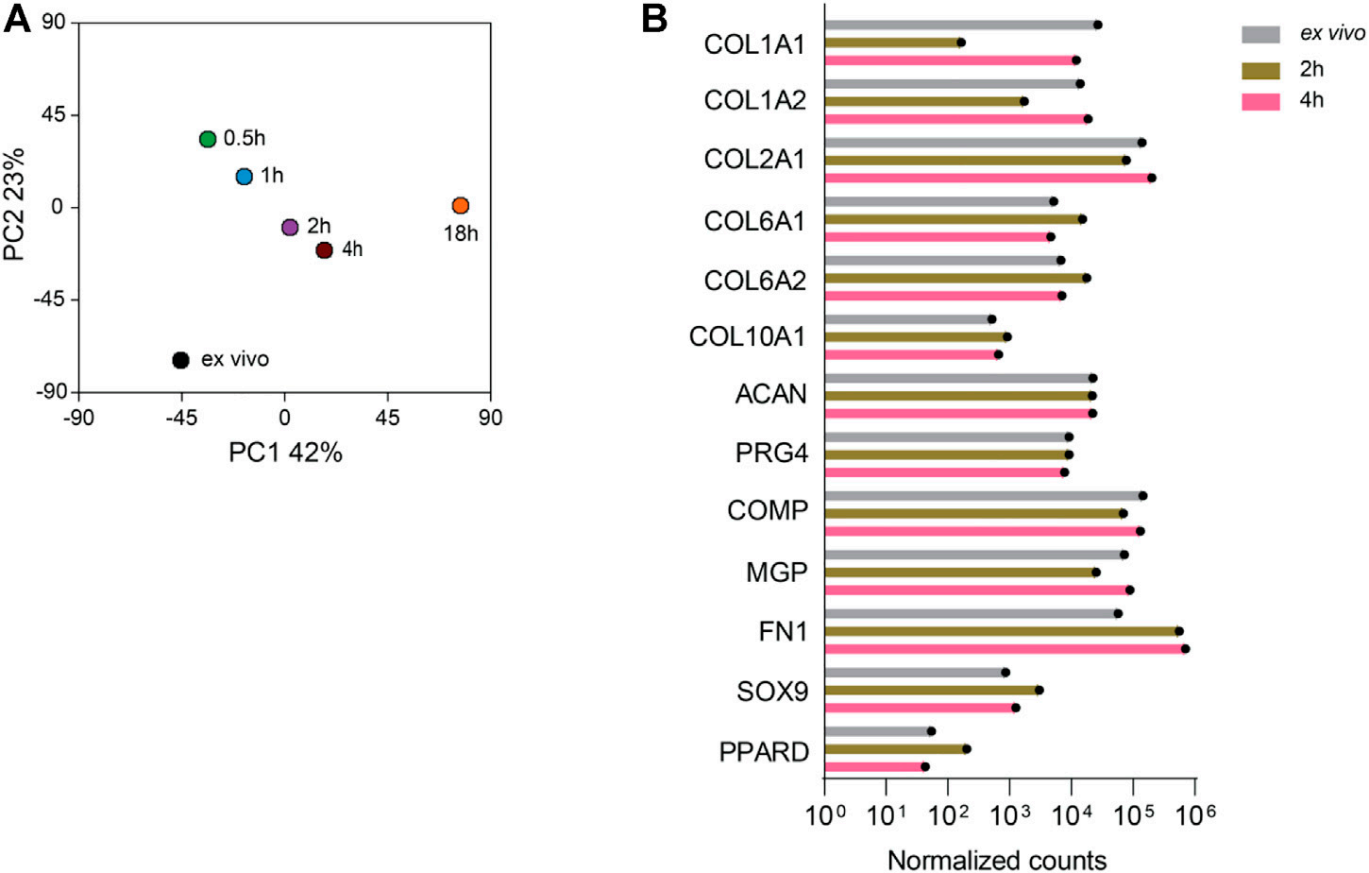
The RNA sequencing profiles of chondrocytes isolated from cartilage by digestion for 2 and 4 h are closest to the RNA-seq profile of the chondrocytes obtained directly ex vivo. RNA-seq analysis was performed on chondrocytes harvested after digestion of cartilage with 2% collagenase II for 0.5, 1, 2, or 4 h and 0.1% collagenase II for 18 h. In addition, RNA was isolated directly ex vivo from the cartilage tissue of the same patient and sequenced. **(A)** The gene expression PCA plot provides a map of the distances between the samples as a measure of the differences between the RNA transcriptomes of the isolated cells and the chondrocytes in cartilage tissue ex vivo. **(B)** The expression of 13 hallmark genes of chondrocytes including COL1A1, COL1A2, COL2A1, COL6A1, COL6A2, COL10A1, ACAN, PRG4, COMP, MGP, FN1, SOX9, and PPARD in the ex vivo sample and after digestion for 2 and 4 h is plotted. Data were generated from the sample from one patient.

We next performed transcriptome analysis of chondrocytes that were obtained by using the digestion protocols of 2%-0.5 h, 2%-1 h, 2%-2 h, 2%-4 h, and 0.1%-18 h. RNA isolated directly from cartilage tissue (*ex vivo*) of the same patient was used as reference. The gene expression principal component analysis (PCA) plot distinguished the cell transcriptomes according to their digestion protocol (Figure 3A): The 0.1%-18 h digestion resulted in a transcriptome that was most distinct from the transcriptome *ex vivo*, as compared to the digestions with reduced incubation time. Thus, shortening the digestion period reduced the transcriptional difference as compared to the *ex vivo* reference. However, it was not simply the shorter, the better, because the 2- and 4-h digestions displayed a closer distance to the *ex vivo* sample than the 0.5- and 1-h digestions when collagenase II was used at a concentration of 2%. To some extent, the transcriptome profiles of the isolated chondrocytes were still different from the *ex vivo* situation. Therefore, we looked at the expression of 13 chondrocyte hallmark genes, including COL1A1, COL1A2, COL2A1, COL6A1, COL6A2, COL10A1, ACAN, PRG4, COMP, MGP, FN1, SOX9, and PPARD in the conditions of *ex vivo*, 2- and 4-h digestions (Figure 3B). We found a similar expression level of the chondrocyte hallmark genes in all three conditions, indicating that the chondrocyte identity seems to be nicely preserved during the 2-h or 4-h isolation period.

### Large chondrocytes require prolonged digestion time for full release from cartilage matrix

**FIGURE 4:**
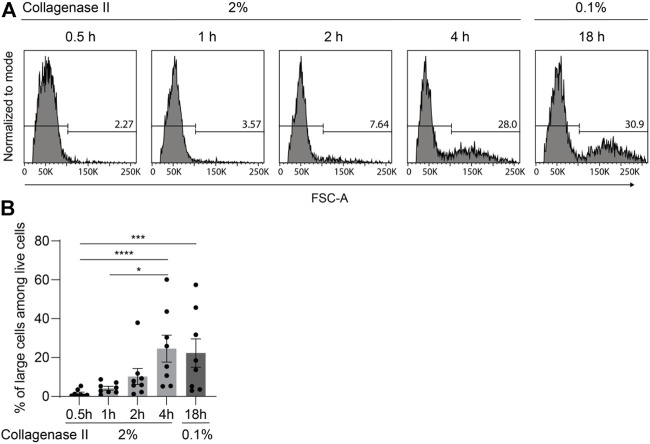
Large chondrocytes require prolonged digestion time for full release from cartilage matrix. After being finely minced, human articular cartilage tissue was divided equally into five portions, and the samples were digested with 2% collagenase II for 0.5, 1, 2, or 4 h; or with 0.1% collagenase II for 18 h. **(A)** Chondrocyte cell size was determined by flow cytometry analysis. Representative FACS histograms and percentages are shown illustrating the cell size with the parameter forward light scatter (FSC-A). **(B)** Percentages of large cells among live cells are plotted (n = 8 patients). Data were analyzed using paired One-way ANOVA (*p < 0.05, ***p < 0.001, ****p < 0.0001).

By using flow cytometry to detect cell viability, we observed a population with larger forward scatter area (FSC-A) indicating large cell size that became more numerous with increasing incubation time (Figure 4A). Notably, the percentage of large cells yielded by the 2%-4 h protocol was around 30%, which is similar to the percentage of large cells obtained by the 0.1%-18 h protocol but substantially higher than the number achieved with the 2%-0.5 h, 2%-1 h, and 2%-2 h protocols (Figure 4B).

### Large chondrocytes do not arise from the small cells during the isolation procedure

**FIGURE 5:**
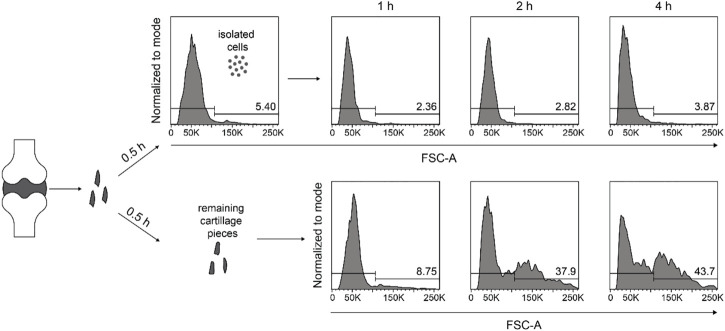
Large chondrocytes do not arise from the small cells during the isolation procedure. Human articular cartilage tissue was minced finely and digested with 2% collagenase II for 0.5 h. Released cells and undigested cartilage tissue were separate by a cell strainer, transferred into two separate tissue culture tubes, and both samples were replenished with medium containing 2% collagenase II, and were kept at 37°C under constant rotation. After another 0.5, 1.5, and 3.5 h of further digestion, both samples were subjected to cell harvesting, and the cell sizes were determined at each harvesting time point by flow cytometry. FACS histograms and percentages from one experiment are shown illustrating the cell size with the parameter forward light scatter (FSC-A).

Next, we determine if the large cells arose from the small cells during the digestion period or if they existed already in the original cartilage tissue but required prolonged incubation time to get released. To this end, we digested the minced cartilage tissue with 2% collagenase II for 0.5 h and separated the cells and remaining cartilage pieces with a cell filter. After replenishing the samples with the same digestion buffer, the cell sizes were determined after a total incubation time of 1, 2, and 4 h. We observed that cells harvested at 0.5 h did not become larger, while the separated cartilage pieces released successively more large cells during the following 3.5 h incubation time (Figure 5). Thus large chondrocytes needed a longer digestion period to get released from cartilage tissue, compared to the small ones.

Notably, the sample from 2-h digestion exhibited a similar distance to the *ex vivo* sample as the 4-h digestion sample. However, given the trend towards a higher proportion of large cells (*cf.* Figure 4B) and a higher yield in the total number of live cells (*cf.* Figure 2A), we considered the 4-h digestion the best protocol among the tested conditions, in terms of transcriptome preservation and cell recovery.

### Down-titration of collagenase II yields optimized chondrocyte recovery including large cells at high viability

**FIGURE 6:**
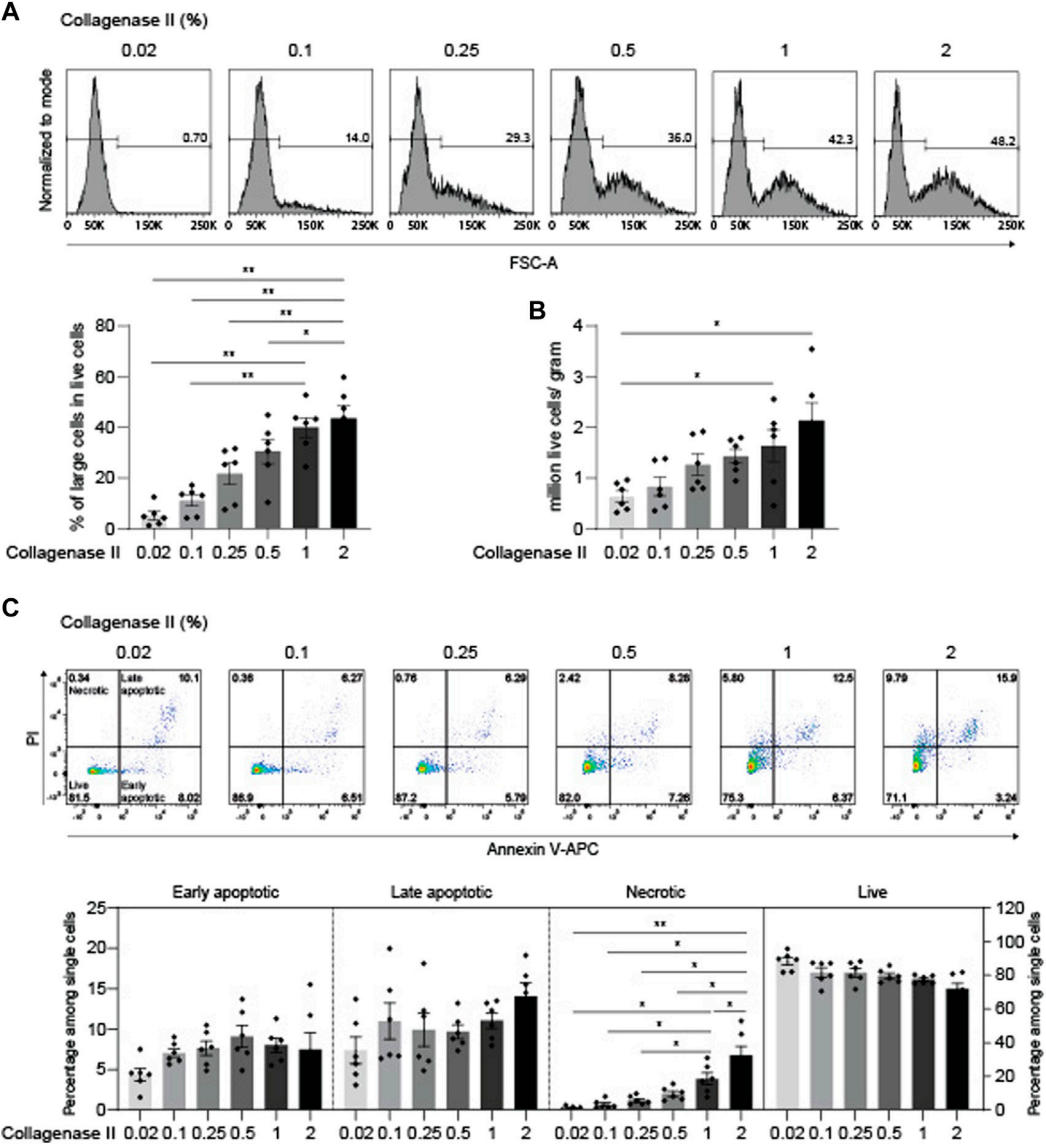
Down-titration of collagenase II yields optimized chondrocyte recovery including large cells at high viability. After being finely minced, human articular cartilage tissue was divided equally into six portions, and the samples were digested for 4 h with 0.02%, 0.1%, 0.25%, 0.5%, 1%, and 2% collagenase II. A collagenase II concentration of 1–2% yields optimal numbers of live chondrocytes including the large cells. **(A)** Chondrocyte cell size was determined by flow cytometry analysis (upper panel). Representative FACS histograms and percentages are shown. Percentages of large cells among live cells (lower panel) and **(B)** total numbers of isolated chondrocytes per gram of cartilage are plotted (n = 6 patients). **(C)** Apoptotic and necrotic states of chondrocytes were determined by propidium iodide (PI) and Annexin V staining (upper panel). Representative dot plots from FACS analysis are shown. Percentages of live, early apoptotic, late apoptotic, and necrotic cells are plotted (lower panel, n = 6 patients). Data were analyzed using paired One-way ANOVA (*p < 0.05, **p < 0.01).

High concentrations of collagenase II can lead to increased apoptosis incidence (Yonenaga et al., 2017). To determine whether chondrocytes tolerate a concentration of collagenase II of 2%, we titrated collagenase II from 0.02% to 2% at a fixed digestion period of 4 h. The percentages of large cells increased gradually with increasing concentrations of collagenase II and reached similar maximal values at 1% and 2% (Figure 6A). This was also the case for the total numbers of recovered live cells (Figure 6B). With regard to apoptosis, including both early and late apoptosis, no significant difference was detected among all the tested conditions, although the 2% concentration was associated with a moderate trend to higher incidences of late apoptosis. However, the percentages of necrotic cells increased gradually with increasing concentrations of collagenase II, and especially the collagenase II concentration of 2% induced a significantly higher frequency of necrosis than the 1% concentration. Nevertheless, the increased but generally still low proportions of necrotic cells had no strong impact on the frequency of live cells, as there was no significant difference in the percentage of live cells among all the tested conditions (Figure 6C). In summary, the digestion protocol of 1%-4 h appeared slightly better than the 2%-4 h condition, as it induced less necrosis and was superior to lower concentrations of collagenase II in releasing large chondrocytes from cartilage tissue.

### Cartilage digestion with 1% or 2% collagenase II for 4 h plus ActD yields a chondrocyte population with highest transcriptome preservation

**FIGURE 7:**
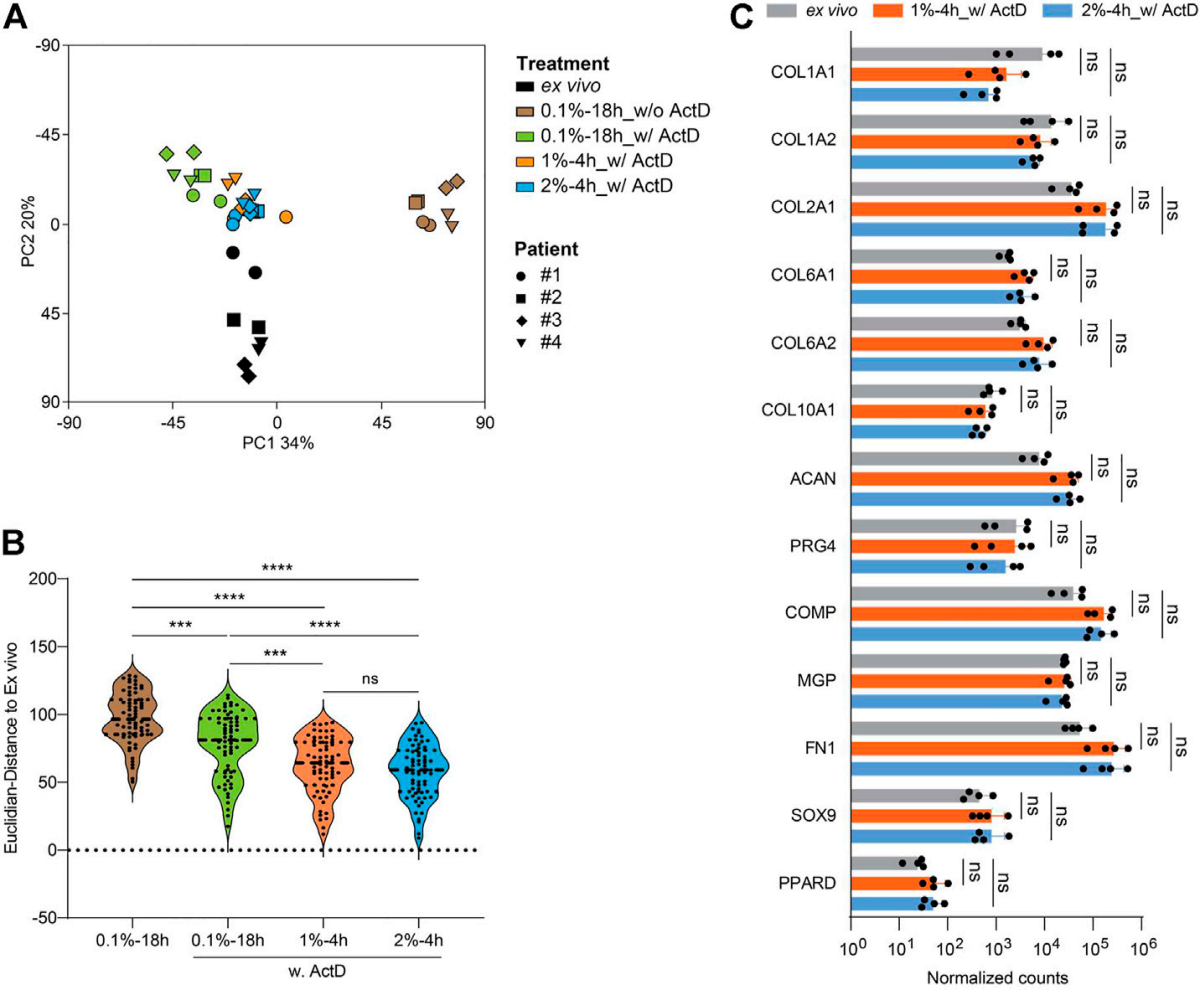
Cartilage digestion with 1% or 2% collagenase II for 4 h plus ActD yields a chondrocyte population with highest transcriptome preservation. RNA-seq analysis was done for chondrocytes harvested after 4-h digestion with 1% or 2% collagenase II, and 18-h digestion with 0.1% collagenase II with or without ActD. In addition, RNA isolated directly ex vivo from cartilage tissue of the same patients was sequenced (each sample two replicates). **(A)** PCA plot shows the clustering of each group of samples. **(B)** The Euclidian distance between the transcriptomes of each group of isolated cells and RNA from total cartilage tissue ex vivo is plotted. **(C)** The expression of 13 hallmark genes of chondrocytes including COL1A1, COL1A2, COL2A1, COL6A1, COL6A2, COL10A1, ACAN, PRG4, COMP, MGP, FN1, SOX9, and PPARD from four patients is plotted. Data in B and C were analyzed using paired One-way ANOVA (ns: *p* > 0.05, ***p < 0.001, ****p < 0.0001).

Next, we performed RNA-seq analysis to determine whether the 1%-4 h digestion protocol can also optimally preserve the transcriptome. Again, we used *ex vivo* RNA of the same patients as positive reference and RNA isolated according to the 0.1%-18 h protocol as negative reference. Actinomycin D (ActD) blocks *de novo* synthesis of RNA transcripts and hence is reported to contribute to the preservation of the RNA transcriptome (Westendorf et al., 2014; Sunkara et al., 2021). Therefore, we added ActD in the following three digestion approaches: 1%-4h, 2%-4h, and 0.1%-18 h. Thus, in total, RNA obtained *via* five protocols was sequenced from each patient: *ex vivo* and 0.1%-18 h without ActD, and 0.1%-18h, 1%-4h, and 2%-4 h each with ActD (Figure 7A). The resulting PCA plot based on global gene expression profiles placed the samples of the protocol 0.1%-18 h without ActD furthest away from the *ex vivo* reference samples and also in large distance from the samples obtained *via* the protocol 0.1%-18 h with ActD, demonstrating that ActD indeed contributed to the stabilization of the RNA transcriptome in chondrocytes during the 18 h digestion period. In the presence of ActD, the distance from the samples of the 1%-4 h or 2%-4 h protocols to the respective *ex vivo* samples was significantly smaller than the distance between the 0.1%-18 h and the *ex vivo* samples. However, the samples obtained *via* the 1%-4h and 2%-4 h protocols largely overlapped and had a similar distance to the respective *ex vivo* samples (Figure 7B). Still, the transcriptome profiles of the isolated chondrocytes were somewhat different from the *ex vivo* situation. Hence, we again compared the expression of 13 chondrocyte hallmark genes in the *ex vivo* samples to the samples obtained after 1%-4 h digestion and 2%-4 h digestion. We did not find a significant difference in the expression of any of these genes, which argues in favor of the maintenance of the chondrocyte characteristics during the isolation procedure (Figure 7C). In conclusion, the 1%-4 h digestion protocol preserved the chondrocyte transcriptome similarly well as the 2%-4 h digestion approach, and these two protocols were significantly better than the 0.1%-18 h digestion condition. Thus, to isolate chondrocytes from human articular cartilage for assays that require optimal maintenance of the *in vivo* transcriptome, a 4-h digestion with 1% or 2% collagenase II plus addition of ActD is recommended.

## Discussion

Numerous protocols for chondrocyte isolation have been described, but almost all of them are aimed at yielding the highest cell recovery and viability, and none of them has been optimized in a systematic way to preserve the chondrocyte transcriptome to the best possible extent. To address this need, we systematically tested various digestion protocols, analyzed the cell size of the isolated chondrocytes, and compared the transcriptome of these cells to the transcriptome obtained from chondrocytes *ex vivo*. We observed that large cells need prolonged digestion time to be released from cartilage tissue, which we found critical for the bulk transcriptome to be representative. Taken together, a 4-h digestion with 1% or 2% collagenase II in the presence of ActD appears to be the optimal protocol to preserve the transcriptome integrity and complexity of chondrocytes isolated from human articular cartilage.

Articular cartilage from adult human knee-joints can be subdivided into a superficial/tangential zone, a middle/transitional zone, and a deep/radial zone. While the cell volume tends to be similar within each individual zone, its mean is reported to increase from about 1,237 μm^3^ in the superficial zone to 2086 μm^3^ in the transitional zone, and to 2,866 μm^3^ in the deep zone (Hunziker et al., 2002). Correspondingly, the mean cell surface area has been calculated to increase from about 672 μm^2^ in the superficial zone to 881 μm^2^ in the transitional zone, and to 1,203 μm^2^ in the deep zone (Hunziker et al., 2002). These features are likely the reason why the large cells embedded in the deep zone, which have an almost two fold larger cell surface area than the small cells in the superficial zone, need longer digestion time to get enzymatically released from their surrounding extracellular matrix. The number of cells, per unit tissue volume, that have a surface area ranging from 600–800 μm^2^, is about 40,838 (=24,018 + 8,879+7,941), and with a surface area larger than 800 μm^2^ it is 24,430 (=10,262 + 7,302+6,866), accounting for around 37% of the total cells in articular cartilage (Hunziker et al., 2002). This number is largely consistent with our flow-cytometry data, and it is in line with a substantial contribution of the large chondrocytes to the transcriptome of the total chondrocyte population in articular cartilage.

To understand the complexity of chondrocyte subpopulations and their gene expression activity, it appears relevant to include the large cells into the RNA-seq analysis. These large cells are embedded preferentially in the deep zone, which predominantly is the remaining zone in articular cartilage from most of the OA patients. Optimal inclusion of large cells into single-cell RNA-seq measurements may increase the resolution of the analysis and hence may allow for the identification of novel subpopulations. Of note, in this study, the chondrocytes were isolated from articular cartilage from OA patients, where a catabolic environment can be assumed. Hence, cartilage from healthy individuals free of OA might need more digestion time or higher concentrations of collagenase II to achieve complete release of all chondrocytes. In addition, cartilage isolated from OA patients with different levels of severity might benefit from individualized conditions to yield an optimal cell release. However, in all cases the mechanical mincing process of the cartilage tissue generates small tissue pieces of macroscopically similar size, which then get exposed to the enzymatic digestion.

The RNA-seq data depicted in Figure 3 suggests that PC1 is largely composed of genes whose expression is influenced by increasing digestion time, while PC2 may rather be comprised of genes expressed by large cells. In the second round of RNA-seq data (*cf.* Figure 7), PC1 appears to arise mainly from genes whose transcription is affected by ActD, and PC2 may rather be generated from gene expression that is affected by the duration of digestion. During a prolonged digestion period, such as 18 h, the usage of ActD to block *de novo* synthesis of RNA transcripts indeed contributed to the preservation of the RNA transcriptome and reduced the deviation from the *ex vivo* situation. However, the necessity of ActD for shorter digestion periods, such as 4 h, was not tested in our current study. Nevertheless, in future studies additional measures to prevent RNA decay might be considered to optimize even further the maintenance of the *ex vivo* transcriptome. It is worth mentioning that RNA isolated directly from cartilage tissue (*ex vivo*) may include the RNA from other cell types residing in the cartilage tissue such as mesenchymal stem cells. Single-cell RNAseq analyses, which may use the improved chondrocyte isolation protocol established in this study, provide the opportunity to distinguish the gene expression of mesenchymal stem cells from the expression profile of chondrocytes.

## Data Availability

The raw data in this article has been deposited in the Gene Expression Omnibus under accession code GSE217871. To review GEO accession GSE217871: Go to https://www.ncbi.nlm.nih.gov/geo/query/acc.cgi?acc=GSE217871.
